# Cross-sectional study of the relationship between trait emotional intelligence and career adaptability of Chinese youths

**DOI:** 10.1186/s12889-023-15372-w

**Published:** 2023-03-17

**Authors:** Hok-Ko Pong, Chi-Hung Leung

**Affiliations:** 1 Faculty of Management and Hospitality Technological and Higher, Education Institute of Hong Kong, Hong Kong, China; 2 Department of Special Education & Counselling, The Education University of Hong Kong, Hong Kong, China

**Keywords:** Trait emotional intelligence, Career adaptability, Chinese youths, University students

## Abstract

**Background:**

Young people often experience dramatic changes, both psychologically and physically, as they are transiting from students to working adults. However, there is still a lack of empirical studies on the relationship between the trait emotional intelligence and the career adaptability of youths in the Asia-Pacific region. This research examines that relationship in Chinese youths in Hong Kong.

**Method:**

Cross-sectional data (*N* = 500) was collected from two universities in 2019 and 2020. The 2019 sample was made up of 256 Chinese university students (117 males, 139 females; ages 21-25). The 2020 sample included 244 Chinese university students (132 males, 112 females; ages 21-25). The participants were asked to complete the Wong and Law Emotional Intelligence Scale (WLEIS) to evaluate their emotional intelligence in the domains of self-emotion appraisal, other people's emotion appraisal, regulation of emotion, and use of emotion. Participants completed the Career Adapt-Abilities Scale (CAAS) to assess their career adaptability in the areas of concern, control, curiosity, and confidence.

**Results:**

All domains of trait emotional intelligence were positively associated with career adaptability. Multiple Regression analysis showed that self-emotion appraisal and appraisal of other people's emotional were the most predictive factors in terms of career adaptability. Together, these two dimensions of emotional intelligence explain 12.5%, 26.2%, 13.4% and 69.4% respectively of the variance in students’ concern, control, curiosity, and confidence in relation to career adaptability.

**Conclusion:**

The results highlight the importance of emotional intelligence in career adaptability. It is thus of value to study further whether career adaptability of young people may improve if emotional intelligence is incorporated into the student curriculum. The findings offer valuable insights for educators and teachers who are responsible for well-rounded development of students, and will thereby foster healthy lifestyles, stable emotional well-being and greater career adaptability in adolescents.

## Introduction

Hong Kong is a leading international financial center [1], in which the pace, transition, and growth of industry can make it difficult for some otherwise outstanding talent to keep up with the demands of this work [2]. The workplace may confront the individual with unpredictable situations, unfamiliar conditions, high demands, and job uncertainty [3, 4]. Young people often undergo tremendous changes both psychologically and physically [5, 6] as they make the transition from student to working adult. The job turnover rate in the first three years after graduation is relatively high [7, 8]. Recognizing the challenges faced in adolescence and young adulthood, Lerner et al [9] indicate that the personal values and environmental assets of young people are important in promoting positive results and enabling them to succeed when encountering adversity [10].

Today, career adaptability—the ability to deal with the constraints of a career and to cope with career changes—is highly important in competitive business societies [11, 12]. Another factor, emotional intelligence, may also be helpful in handling career transitions [13]. The beneficial role of emotional intelligence has been empirically well documented with regard to career decision-making [14, 15], career commitment [16], career success [17] and employability [18]. Emotional intelligence and career adaptability are psychosocial meta-capacities that facilitate successful adaptation in various spheres of life, including that of career [4, 19]. Nevertheless, little is known about the relationship between emotional intelligence and career adaptability [12, 19], especially in youths and in the Chinese social context, such as that encountered Hong Kong.

Accordingly, we addressed the following research questions with a sample of youths from universities in Hong Kong. Is emotional intelligence associated with career adaptability? Are these associations equally strong for different dimensions of career adaptability and different domains of emotional intelligence?

## Definitions and models of emotional intelligence (EI)

Emotional intelligence (EI) has been studied extensively in both western countries [20–24] and in eastern countries [25–27]. Different schools of research adopt different conceptualizations and measures of emotional intelligence [28]. However, EI was a relatively new concept and idea in 1990s before Salovey and Mayer [29] formally introduced the first model and definition. Later, studies have rushed to develop and construct measures of EI, but have ignored the fundamental difference between typical versus maximal performance, which has led to confusing and conflicting results for trait EI and EI [30–32].

Petrides and Furnham [33] clearly articulate a conceptual distinction between trait EI (or emotional self-efficacy) and ability EI (or cognitive-emotional ability). For example, trait EI measured by self-report questionnaires is not expected to correlate strongly with measures of general cognitive ability or its proxies. Whereas ability EI measured through tests of maximal performance, should be clearly related to such cognitive ability measures. This is despite the fact that trait EI despite is theoretically supported by the ability EI paradigm [30, 32]. This measurement difference has profound theoretical and practical implications. Ability EI is a product of one’s mental abilities [29]. The most accepted competency-based model of EI is the four-branch hierarchical model, which divides emotional intelligence into four narrow competencies. It refers to how indivduals reasons about their own emotions, including perceiving them, using them to facilitate thinking, understanding their meaning, and managing their own and others' emotions [29]. Therefore, ability emotional intelligence is a broad competence as it involves the cognitive processing of emotions and emotional information.

This term Trait EI refers to the perception and understanding of one's own and others' emotions; reasoning and judgment about these emotions; and the ability to properly adjust and manage those emotions [29]. It also includes the ability to access and express feelings when beneficial [29] and appropriate [34]. These traits make up a significant inner resource for adaptive intrapersonal and interpersonal relationships [29]. They are also associated with higher life adaptability and a lower risk of suicide [35], higher academic achievement [36], and better social relations [29, 37]. The current study examines trait emotional intelligence in relation to another form of adjustment, namely career adaptability.

Emotional intelligence is thought to be closely related to other forms of intelligence that may help to promote career adaptability. Emotion is integral to thinking and reasoning [21, 38], and the ability to control feelings may promote intellectual development [29]. Emotional intelligence may also be important in enabling individulas to perform to their full potential [39]. Some young people score highly on tests of cognitive ability but achieve little, while others have average scores but achieve extraordinary things [40]. The reason may for this may lie in emotion-related traits and behaviors such as self-control, enthusiasm, perseverance, and self-drive [40]. Studies in the past decades indicate that in terms of measurement most achievements are related to trait EI rather than ability EI [30]. In one study, positive emotional traits were found to be more predictive of future success or failure than scores on tests of cognitive ability [40].

In this study we used the Wong and Law Emotional Intelligence Scale [27] to assess emotional intelligence. This self-report instrument is based on the conceptualization of EI presented by Salovey & Mayer [29]. Wong & Law [27] conceptualize EI as having four distinct dimensions (SEA, OEA, ROE, and UOE), and Wong documented that the WLEIS had a robust four-factor structure. *Self-emotion appraisal* [SEA] is the ability of individuals to understand their feelings and express their emotions as expected. Individuals competent in this dimension feel and perceive their emotions earlier than do most people. *Other people's emotion appraisal* [OEA] is the ability to perceive and understand the emotions of others. Individuals with this ability are more sensitive to the feelings and emotions of others than most people. *Regulation of emotion* [ROE] is the ability to monitor, evaluate, and act to change our own emotions. Individuals with this ability are more able to modify their emotional responses. *Use of emotion* [UOE] is the ability to employ their emotions by directing them toward productive actions and performance. Individuals with this ability are more able to make good use of emotional feelings.

### Definitions and models of career adaptability

Super & Knasel [41] describe career adaptability as an individual's state of preparedness to respond to changes in work. For workers to adapt and survive, they must show flexibility in adjusting suitably to ongoing change [42–44]. Career adaptability can be thought of as an interaction between the individual and a constantly changing environment. Environmental changes include not only shifts in economic forces and job markets, but also changes in the workplace, including changes in coworkers [43].

There have been decades of research on workers' decisions to change jobs, careers, or even the field in which they work [43]. Workers who show career adaptability use purposeful, flexible strategies to meet and respond to changes in a complex business environment [45]. Compared to other workers, they are usually more ambitious and self-motivated about their careers, more sensitive to the environment, and more aware of changes in the organization and the industry [41]. In addition, career adaptability has been shown to be correlated with education level, the professionalism of the occupation, and work status. People with these advantages may have more flexible roles and communication skills, and more opportunities to make career adjustments [46].

Savickas [43, 45] proposes three overarching domains of career adaptability: exploration, prudent planning, and adaptive decisions. In this structural model, these three overarching domains can be divided into four lower order dimensions, namely career concern, career control, career curiosity and career confidence [44]. These four constructs represent universal resources and strategies to face career tasks, job changes, or major events, as individuals construct their careers [43]. In the current research we used a measure of career adaptability based on this model. 

The Career Adapt-Abilities Scale (CAAS)—China Form [47] is a self-reported career adaptability scale based on Savickas & Porfeli's [48] conceptual framework. Hou et al. [47] confirm that the CAAS China Form is identical in contexts to the International Form, including having a four-domain structure. More recent research confirms the four-factor structure [49]. The measure, consistent with the conceptual model has four subscales. *Career concern* (concern): the individual's concern for their future development, and awareness of the importance of preparing for this development. *Career control* (control): the individual's sense of control over their future development and the belief that they can take responsibility for a self-constructed career. *Career curiosity* (curiosity): individual's curiosity about and anticipation of the possibility of self-development in the future, and willingness to actively explore this possibility. *Career confidence* (confidence): individual's confidence in overcoming difficulties that will be encountered in the development of the self, including a sense of self-efficacy about whether they can handle the self properly.

### Research on emotional intelligence and career adaptability

In the past decade, there have been studies, such as studies of Coetzee and Harry [19] in Africa, Parmentier et al. [4] in Belgium, and Udayar et al. [50] in Switzerland for the relationship between emotional intelligence and career adaptability. Udayar et al., [50] showed that, controlled by their intelligence, sex and personality, participants’ career adaptability facilitates the effect of Trait Emotional intelligence on self-perceived employability and career decision-making difficulties, especially when information is unreliable.

Coetzee and Harry [19] finds in their cross-sectional survey, via its sample of 409 early career black center agents, that managing one's own emotions contributes most to explaining overall emotional intelligence and the variance in overall career adaptability with its four domains, including concern, control, confidence and curiosity. The two-wave longitudinal study of Parmentier, Pirsoul, and Nils [4] from a sample of 282 adult learners, and controlling for prior levels of career adaptability and socio-demographic variables, finds that emotional intelligence at Time 1 predicts career adaptability at Time 2.

### Present study

The current study tests the relation between emotional intelligence and career adaptability among Chinese youths in Hong Kong. A cross-sectional survey was completed by 500 year-4 (final year) university students. This research will add to the current literature by examining the relationship between multiple dimensions of emotional intelligence and multiple dimensions of career adaptability as part of a broader set of youth resources and outcomes among Chinese university students.

### The aims and hypothesis

According to Cobb and Mayer [51] and Savikkas and Porfeli [48], both paradigms are self-regulatory approaches and abilities in human being, as important psychological assets and social interactions. Based on the career construction model [43], some empirical studies have demonstrated emotional intelligence’s positive effect on career adaptability. This current study examines the relationship between emotional intelligence and career adaptability in youths in Asia-Pacific regions, especially in the Chinese cultural context, based on the above previous empirical studies. It is expected that emotional intelligence positively predicts career adaptability.

## Method

### Participants

Five hundred Chinese students from two universities in Hong Kong participated either in 2019 (*n* = 256) or 2020 (*n* = 244). X and Y are used as the names of the two selected universities. The young participants were studying for Business major discipline at these two universities. All were all local Chinese, educated and raised in Hong Kong. Year 4 students were the targeted participants because they were soon to complete the 4-year bachelor's degree and graduate.

One of the focuses of the study is career adaptability. It was considered suitable to recruit business students as participants for the research as they will work in a wide variety of industries and in different kinds of jobs after graduation. The range of careers is very diverse. Students were recruited from these two universities because they are the universities that offer the most places to study business in Hong Kong, and thus have the most business students. It was easier to find suitable and sufficient research samples. The study recruited year-4 business students because they were relatively less affected by other factors than other working Chinese youth. This would be more objective for the analysis in the study.

Power analysis showed that a sample of 500 with small, medium, and large effect size of .32, .57 and .65 respectively, was enough to generate a precise estimate of the regression coefficient with a small confidence interval around each estimate.

The 2019 sample consisted of 256 Chinese university students (117 males, 139 females; ages 21-25) about half of whom (51.95%; *n* = 133) came from University X and about half (48.05%; *n* = 123) from University Y. The 2020 sample included 244 Chinese university students (132 males, 112 females; ages 21-25). About half of whom (47.54%; *n* = 116) came from University X and the rest (52.46%; *n* = 128) from University Y.

A series of t-tests found no significant mean differences between the 2019 and 2020 samples on the total scores or the dimension scores of the measures of emotional intelligence and career adaptability (*p* > .05). Thus, the samples from 2019 and 2020 were combined in the analyses.

### Measures

Participants were allowed to complete the measures in Chinese or English. Both measures used in the study were Chinese language measures. We used the translation and back-translation method to translate these measures to English.

### Demographics

Participants provided demographic information about gender, age, university.

### Emotional intelligence: Wong and Law Emotional Intelligence Scale (WLEIS)

On the Wong and Law Emotional Intelligence Scale [27] participants rate how much they agree with each of 16 statements, using a five-point Likert scale (1 = *strongly disagree* to 5 = *strongly agree*). The measure was employed to measure four aspects of emotional intelligence: self-emotion appraisal (SEA; 4 items, e.g., “I have good understanding of my own emotions”), others’ emotion appraisal (OEA; 4 items, e.g., “I have good understanding of the emotions of people around me”), use of emotion (UOE; 4 items, e.g., “I always set goals for myself and then try my best to achieve them”), and regulation of emotion (ROE; 4 items, e.g., “I can always calm down quickly when I am very angry”). The overall score and the subscale scores are calculated as means. The overall scale showed acceptable internal consistency, with Cronbach's alpha = .86. The alpha values for the SEA, OEA, UOE, and ROE subscales were also acceptable at .75, .94, .78, and .72, respectively. This measure has been developed and validated in the Chinese cultural context in samples of Chinese individuals from Mainland China [52] and Hong Kong [27].

Wong showed that the WLEIS had a robust four-domain structure. In the current study, Principal Components Analysis indicates that this structure is also appropriate for use in our sample (Kaiser-Meyer-Olkin value = 0.92; Bartlett’s test of sphericity significant at *p <* .001). Exploratory Factor Analysis identifies four factors (each with an eigenvalue *>* 1.0) corresponding to the OEA, UOE, ROE, and SEA domains. These four subscales explained 35.75%, 15.35%, 10.75%, and 7.06%, of the variance, respectively. Table 1 shows the factor loadings for the four-factor model in the current sample.

**Table 1:** Results of Exploratory Factor Analysis of the Wong and Law Emotional Intelligence Scale WLEIS items (*N* = 500)

**Rotated Component Matrix**^**a**^				
	Component			
	OEA	UOE	ROE	SEA
1. I have a good sense of why I have certain feelings most of the time.	.17	.04	.11	**.75**
2. I have good understanding of my own emotions.	.11	.14	.10	**.77**
3. I really understand what I feel.	.00	.22	.36	**.62**
4. I always know whether or not I am happy.	.03	.19	.28	**.68**
5. I always know my friends’ emotions from their behavior.	**.88**	.06	.13	.11
6. I am a good observer of others’ emotions.	**.88**	.16	.07	.09
7. I am sensitive to the feelings and emotions of others.	**.90**	.14	.15	.08
8. I have good understanding of the emotions of people around me.	**.92**	.12	.10	.07
9. I always set goals for myself and then try my best to achieve them.	.06	**.83**	.07	.12
10. I always tell myself I am a competent person.	.39	**.57**	-.10	.18
11. I am a self-motivated person.	.10	**.86**	.13	.06
12. I would always encourage myself to try my best.	.14	**.69**	.22	.23
13. I am able to control my temper and handle difficulties rationally.	.14	.03	**.66**	.19
14. I am quite capable of controlling my own emotions.	-.02	.24	**.46**	.25
15. I can always calm down quickly when I am very angry.	.14	.09	**.72**	.13
16. I have good control of my own emotions.	.11	.02	**.91**	.13

Participants were asked to complete the Wong and Law Emotional Intelligence Scale WLEIS items [27], 16 items divided equally into four subscales measuring the s*elf-emotion appraisal* [SEA], other people's emotion appraisal [OEA], regulation of emotion [ROE] and use of emotion [UOE]

### Career Adaptability: The Career Adapt-Abilities Scale (CAAS) – China Form

The Career Adapt-Abilities Scale – Chinese Form [47] is a 24-item self-report measure used to measure individuals’ responses to changes in the career by the use of methods or strategies. How strong is your dedication to developing the following characteristics? Items are rated on a 5-point Likert scale (1 = *not strong* to 5 = *strongest*) and ratings are averaged to create an overall scale and four subscales: concern (6 items; e.g., “concerned about my career”), control (6 items; e.g., “making decisions by myself"), curiosity (6 items; e.g., “becoming curious about new opportunities”), and confidence (6 items; e.g., “overcoming obstacles”). These subscales had acceptable internal consistency in the current sample, with Cronbach alphas = .96, .98, .98, and .985, respectively; for the overall scale, alpha = .96.

Principal Components Analysis showed that the four-domain structure identified in earlier research such as that of Hui et al. [49] and Ye [53], was appropriate for use in our sample (Kaiser–Meyer–Olkin value = .92; Bartlett’s test of sphericity significant at *p <* .001). Exploratory Factor Analysis identified four factors (each with an eigenvalue *>* 1.0) corresponding to the confidence, curiosity, concern, and control domains. These factors explained 52.80%, 17.34%, 12.50%, and 7.18%, of the variance, respectively. Table 2 shows the factor loadings for the four-factor model in the current sample.

**Table 2:** Results of Exploratory Factor Analysis of items on the Career Adapt-Abilities Scale – Chinese (*N* = 500)

**Rotated Component Matrix**^**a**^				
	Component			
	Confidence	Curiosity	Concern	Control
1. Thinking about what my future will be like	.13	.14	**.83**	.36
2. Realizing that today's choices shape my future	.11	.17	**.89**	.23
3. Preparing for the future	.14	.15	**.86**	.29
4. Becoming aware of the educational and career choices that I must make	.08	.23	**.87**	.24
5. Planning how to achieve my goals	.13	.26	**.81**	.19
6. Concerned about my career	.15	.18	**.87**	.25
7. Keeping upbeat	.21	.28	.26	**.85**
8. Making decisions by myself	.27	.27	.36	**.76**
9. Taking responsibility for my actions	.25	.29	.30	**.81**
10. Sticking up for my beliefs	.27	.12	.33	**.83**
11. Counting on myself	.24	.27	.31	**.83**
12. Doing what's right for me	.28	.21	.31	**.84**
13. Exploring my surroundings	.06	**.89**	.20	.23
14. Looking for opportunities to grow as a person	.15	**.90**	.12	.21
15. Investigating options before making a choice	.06	**.91**	.14	.21
16. Observing different ways of doing things	.12	**.92**	.18	.20
17. Probing deeply into questions I have	.08	**.89**	.21	.17
18. Becoming curious about new opportunities	.14	**.91**	.25	.11
19. Performing tasks efficiently	**.95**	.11	.04	.21
20. Taking care to do things well	**.93**	.15	.13	.18
21. Learning new skills	**.93**	.14	.14	.19
22. Working up to my ability	**.92**	.10	.20	.18
23. Overcoming obstacles	**.93**	.07	.09	.21
24. Solving problems	**.93**	.05	.12	.22

Participants were asked to complete the Chinese version of the Career Adapt-Abilities Scale [47], which consists of 24 items divided equally into four subscales measuring the adaptive resources of concern, control, curiosity and confidence

### Procedure

Approval to conduct this study was obtained from the Research Ethics Committee of the institution with which the first author is affiliated. Convenient and snowballing sampling were employed. The students were recruited with the help of lecturers, educational administrators, and program leaders from the two universities. The data was collected through the cross-sectional survey in the academic years of 2018-2019 (paper-and-pencil questionnaires) and 2019 – 2020 (online questionnaires via electronic Google form). There was a declaration statement on the front page of the questionnaire. The anonymity of all participants was guaranteed. A total of 500 out of 750 Chinese students from the two universities in Hong Kong completed the self-administered, anonymous and confidential questionnaires in both surveys, a response rate of 66.7%.

The questionnaire study took 15 minutes to complete. These students were told the purpose of the study and informed that participation was voluntarily, that they could withdraw at any time without penalty or prejudice, that no compensation was offered, and that all information was confidential. Participants provided informed consent in writing.

Due to the bilingual cultural context of Hong Kong, the questionnaires were presented in Chinese and English. Although participants could complete either version. ll the questionnaires were completed in Chinese. For the academic year 2018-2019, the participants were given paper-and-pencil questionnaires in the classroom, which they returned to the course instructor. Students who chose not to participate would be allowed to leave the classroom. The research started on March 4, 2019 and ended on March 15, 2019.

For the academic year 2019-2020, due to the Covid-19 pandemic, an online survey was used. Emails were sent to the targeted participants with the help of lecturers and program leaders. They contained a hyperlink to an online survey website that included an informed consent form and questionnaires. Browser cookies were set up to prevent respondents from using the same browser to participate in the survey a second time. This design can prevent repeated responses. The online survey started on March 2, 2020 and ended on 31 March 2020.

## Results

SPSS Version 26 was adopted for data analysis. Data cleaning was performed to correct coding errors and illogical data values. There was no missing data because participants had enough time to complete the questionnaire and were given a brief introduction before taking the survey so that they fully understood the included question items. Also, most participants were students of the authors, so they approached the tasks seriously thanks to good teacher-student relationships. Visual inspection of the distribution of data sets was used for assessing normality. We judged the distribution of each data sets and examined the frequency distributions. The outcomes showed a bell shape curves distribution of the data sets.

### Descriptive statistics

Table 3 shows the descriptive statistics as well as the results of analyses examining potential demographic differences on the CAAS (concern, control, curiosity, confidence, and overall score) and WLEIS (SEA, AOE, UOE, ROE, and overall score). Similarly, *t* tests found no significant differences on these scores based on sample year (2018-2019, 2019-2020) or university (University X, University Y). Most of participants belonged to the same group based on socioeconomic status, in terms of their annual household income and highest level of parental education. The monthly incomes of the majority (85%) of participants’ households were between HK$38,001 and HK$58,000, higher than the median monthly household income (HK$28,300 in 2018). The parents of the participants had at least secondary education, and 60% and 50% of the participants' fathers and mothers, respectively, had a post-secondary education level or above as shown in table 4. Most of them belong to the middle class in Hong Kong. Thus, the sociodemographic background of the participants did not differ significantly.

**Table 3:** Descriptive statistics: participants’ demographics and their relationship with emotional intelligence and career adaptability (*N* = 500)

Factors	N (%)		SEA *M* (*SD*)	OEA *M* (*SD*)		UOE *M* (*SD*)		ROE *M* (*SD*)		WLEIS overall *M* (*SD*)		Concern *M* (*SD*)		Control *M* (*SD*)		Curiosity *M* (*SD*)		Confidence *M* (*SD*)	CAAS Overall *M* (*SD*)
All	500 (100%)		4.10 (.53)	3.12 (1.06)		3.53 (.69)		3.73 (.47)		3.62 (.50)		3.70 (.55)		3.52 (.55)		2.75 (.75)		3.71 (.81)	3.42 (.50)
Year of data collection																			
2019	256 (51.2%)		4.10 (.54)	3.11 (1.05)		3.57 (.70)		3.73 (.48)		3.63 (.50)		3.69 (.55)		3.52 (.55)		2.74 (.75)		3.72 (.81)	3.42 (.50)
2020	244 (48.8%)		4.10 (.53)	3.13 (1.06)		3.49 (.69)		3.73 (.48)		3.61 (.49)		3.72 (.54)		3.52 (.56)		2.77 (.75)		3.70 (.81)	3.43 (.51)
			*t = .03*	*t = -.22*		*t = 1.55*		*t = -.07*		*t = .41*		*t = -.07*		*t = .14*		*t = -.54*		*t = .17*	*t = -.27*
Age																			
22 and below	134 (26.8%)		4.04 .52	3.06 1.07		3.55 .70		3.67 .51		3.58 .50		3.73 .55		3.45 .58		2.63 .76		3.68 .83	3.37 .51
23	160 (32%)		4.13 .55	3.17 1.09		3.54 .72		3.75 .46		3.65 .51		3.68 .55		3.53 .55		2.89 .76		3.75 .79	3.46 .51
24	119 (23.8%)		4.11 .52	3.13 1.04		3.44 .62		3.78 .46		3.62 .47		3.64 .56		3.56 .56		2.77 .72		3.72 .81	3.42 .51
25	87 (17.4%)		4.11 .52	3.09 1.03		3.59 .70		3.73 .46		3.63 .49		3.80 .51		3.56 .53		2.67 .69		3.68 .81	3.43 .48
			*F* (3, 499) = .70	*F* (4, 499) =.29		*F* (4, 499) =.10		*F* (4, 499) =1.24		*F* (4, 499) =.48		*F* (4, 499) =1.70		*F* (4, 499) =1.07		*F* (4, 499) =3.59		*F* (4, 499) =.25	*F* (4, 499) =.80
Gender																			
Male	249 (49.8%)		3.98 (.48)	3.05 (1.02)		3.50 (.70)		3.67 (.46)		3.55 (.48)		3.67 (.58)		3.44 (.56)		2.64 (.73)		3.56 (.76)	3.33 (.48)
Female	251 (50.2%)		4.22 (0.56)	3.18 (1.09)		3.56 (.69)		3.80 (.47)		3.69 (.50)		3.74 (.52)		3.60 (.54)		2.87 (.75)		3.86 (.82)	3.52 (.51)
			t =-5.04**	*t =-1.38*		*t =-.95*		t =-3.16*		t =-3.16*		*t =-1.30*		t =-3.25*		t =-3.42*		t =-4.34**	t =-4.28**
Universities																			
X	249 (49.8%)		4.10 (.55)	3.10 (1.08)		3.50 (.71)		3.74 (.48)		3.61 (.51)		3.73 (.53)		3.52 (.57)		2.76 (.74)		3.72 (.82)	3.43 (.51)
Y	251 (50.2%)		4.10 (.52)	3.14 (1.04)		3.55 (.68)		3.73 (.47)		3.63 (.48)		3.68 (.56)		3.52 (.54)		2.75 (.75)		3.70 (.80)	3.41 (.50)
			*t =-.19*	*t = -.44*		*t = -.76*		*t =.19*		*t =-.51*		*t =.99*		*t =-.13*		*t =.25*		*t =.32*	*t =.45*
Religious beliefs																			
With	142	4.44		3.62	3.77		3.94		3.94		3.84		3.71		2.94		4.23		3.68
	(28.4%)	(.44)		(.95)	(.67)		(.39)		(.40)		(.47)		(.47)		(.75)		(.72)		(.42)
Without	358	3.97		2.92	3.43		3.65		3.49		3.65		3.45		2.68		3.51		3.32
	(71.6%)	(.51)		(1.03)	(.68)		(.48)		(.47)		(.57)		(.57)		(.73)		(.74)		(.50)
	*t* = 9.65**			*t* = 6.99**	*t* = 5.16**		*t* = 6.40**		*t* = 10.05**		*t* = 3.41*		*t* = 4.91**		*t* = 3.52**		*t* = 9.93**		*t* = 7.56**

*CAAS* Career adapt-abilities scale. The CAAS subscales are concern, control, curiosity, and confidence, *WLEIS* Wong and law emotional intelligence scale, The WLEIS subscales are *SEA* Self-emotion appraisal, *OEA* Other people's emotion appraisal, *UOE* Use of emotion, *ROE* Regulation of emotion

*N* = 500

^*^p < 0.01. **p < 0.001

**Table 4:** Statistics: Monthly household income and parental highest education level

**Monthly family income**	**N (%)**	**Cumulative Percentage**
1.Below HKD$28,300	15 (3%)	3
2.From HKD$28,300 to HKD$38,000	35 (7%)	10%
3.From HKD$38,001 to HKD$48,000	225 (45%)	55%
4.From HKD$48,001 to HKD$58,000	200 (40%)	95%
5.Above HKD$58,000	25 (5%)	100%
**Remark: HK$ 7.78 = US$ 1**		
**Father’s highest education level**	**N (%)**	**Cumulative Percentage**
1.Secondary education level	200 (40%)	40%
2.Tertiary education level, including diploma, associate degree, and bachelor’s degree	250 (50%)	90%
3.Postgraduate education level	50 (10%)	100%
**Mother’s highest education level**	**N (%)**	**Cumulative Percentage**
1.Secondary education level	250 (50%)	50%
2.Tertiary education level, including diploma, associate degree, and bachelor’s degree	225 (45%)	90%
3.Postgraduate education level	25 (5%)	100%

Table 5 shows the correlations between scores on the CAAS and scores on the WLEIS. There was a statistically significant relationship between graduates’ emotional intelligence in all domains (i.e., SEA, OEA, UOE and ROE) and all areas of career adaptability (i.e., concern, control, curiosity and confidence). Relations involving OEA, UOE, and ROE were small to moderate (Pearson’s *r* values from .17 to .42) while relations involving the SEA domain were near moderate to strong (.28 to .83). The findings indicate that the higher the emotional intelligence score on each domain, the higher the score on each career adaptability subscale.

**Table 5:** Pearson correlations between scores on the Wong and Law Emotional Intelligence Scale and scores on the Career Adapt-Ability Scale (*N* = 500)

	WLEIS SEA	WLEIS OEA	WLEIS UOE	WLEIS ROE	WLEIS Overall
CAAS Concern	.33**	.22**	.17**	.19**	.31**
CAAS Control	.48**	.30**	.24**	.30**	.44**
CAAS Curiosity	.28**	.30**	.15**	.18**	.33**
CAAS Confidence	.83**	.27**	.39**	.42**	.61**
CAAS Overall	.66**	.36**	.32**	.37**	.57**

*CAAS* Career adapt-abilities scale, The CAAS subscales are Concern, Control, Curiosity, and Confidence, *WLEIS* Wong and law emotional intelligence scale. The WLEIS subscales are *SEA* Self-emotion appraisal, *OEA* Other people's emotion appraisal, *UOE* Use of emotion, *ROE* Regulation of emotion

*N* = 500

^*^p < .05. **p < .01. ***p < .001

Multiple regression analyses were conducted with the demographic variables that showed significant influence, together with students’ emotional intelligence domains as predictor variables for concern, control, curiosity and confidence of career adaptability as dependent variables in four analyses.

Multiple regressions were used to predict each CAAS score. SEA and OEA were entered into model 1 and 2 respectively as predictors. UOE and ROE were added to Model 3 and Model 4, respectively to predict each CASS, as shown in Table 6. Then, the demographic variables that revealed significant influence, such as gender and religious beliefs, were entered into Model 5 and Model 6 respectively as predictors as well.

**Table 6:** Results of multiple regression analyses with the demographic variables and emotional intelligence in the SEA, OEA, UOE and ROE domains of the Wong and law emotional intelligence scale as predictors of the concern, control, curiosity, and confidence domains of the career adapt-abilities scale

Predicted	Predictor								
CASS – concern		Standardized Coefficients Beta	*B*	*T*	*F*	*R*	*R*^*2*^	*ΔR*^*2*^	*Adjusted R*^*2*^
Model 1				13.07	58.64	.33	.11	.11	.10
	SEA	.33	.33						
Model 2					35.59	.35	.13	.02	.12
	SEA	.29	.29	6.64					
	OEA	.15	.08	3.37					
Model 3					23.68	.35	.13	0	.12
	SEA	.29	.30	6.23					
	OEA	.15	.08	3.23					
	UOE	.00	.00	-.05					
Model 4					17.73	.35	.13	0	.12
	SEA	.29	.30	5.61					
	OEA	.15	.08	3.19					
	UOE	.00	.00	-.05					
	ROE	.00	.00	-.02					
Model 5					14.18	.35	.13	0	.12
	SEA	.29	.30	5.57					
	OEA	.15	.80	3.19					
	UOE	.00	.00	-.07					
	ROE Gender	.00 -.02	.00 -.02	.00 -.35					
Model 6	SEA OEA UOE ROE Gender Religious beliefs	.30 .15 .00 .00 -.02 .01	.30 .08 .00 .00 .00 .01	5.41 3.16 -.06 .01 -.34 .18	11.80	.35	.13	0	.12
CASS – Control		Standardized Coefficients Beta	*B*	*T*	*F*	*R*	*R*^*2*^	*ΔR*^*2*^	*Adjusted R*^*2*^
Model 1					147.43	.48	.23	.23	.23
	SEA	.48	.50	12.14					
Model 2					88.05	.51	.26	.03	.26
	SEA	.43	.45	10.81					
	OEA	.19	.10	4.73					
Model 3					58.59	.51	.26	0	.26
	SEA	.43	.45	10.16					
	OEA	.19	.10	4.55					
	UOE	-.01	.00	-.12					
Model 4					44.19	.51	.26	0	.26
	SEA	.41	.43	8.71					
	OEA	.18	.10	4.35					
	UOE	-.01	-.01	-.17					
	ROE	.05	.05	1.00					
Model 5					35.54	.51	.27	0	.26
	SEA	.40	.42	8.39					
	OEA	.18	.10	4.33					
	UOE	-.01	.00	-.11					
	ROE Gender	.04 .04	.052 .042	.97 .97					
Model 6	SEA OEA UOE ROE Gender Religious beliefs	.41 .19 -.01 .05 .04 .02	.42 .10 .00 .05 .04 .02	8.19 4.33 -.11 .99 .97 .43	29.60	.51	.27	0	.26
CASS – Curiosity		Standardized Coefficients Beta	*B*	*T*	*F*	*R*	*R*^*2*^	*ΔR*^*2*^	*Adjusted R*^*2*^
Model 1					42.82	.28	.08	.08	.08
	SEA	.28	.39	6.54					
Model 2					38.46	.37	.13	.06	.13
	SEA	.22	.31	5.12					
	OEA	.24	.17	5.61					
Model 3					25.83	.37	.14	0	.13
	SEA	.23	.33	5.07					
	OEA	.25	.18	5.60					
	UOE	-.04	-.04	-.80					
Model 4					19.34	.37	.14	0	.13
	SEA	.23	.33	4.57					
	OEA	.25	.18	5.53					
	UOE	-.04	-.04	-.79					
	ROE	.00	.00	-.04					
Model 5									
	SEA	.21	.30	4.12	16.49	.38	.14	0	.13
	OEA	.25	.18	5.51					
	UOE	-.03	-.04	-.67					
	ROE Gender	-.01 .09	-.01 .14	-.11 2.13					
Model 6	SEA OEA UOE ROE Gender Religious beliefs	.22 .25 -.03 -.01 .09 .01	.30 .18 -.04 -.01 .14 .01	4.01 5.42 -.67 -.11 2.13 .14	13.71	.38	.14	0	.13
CASS – Confidence		Standardized Coefficients Beta	*B*	*T*	*F*	*R*	*R*^*2*^	*ΔR*^*2*^	*Adjusted R*^*2*^
Model 1					1107.67	.83	.69	.69	.69
	SEA	.83	1.25	33.28					
Model 2					564.33	.83	.69	.004	.69
	SEA	.81	1.23	31.74					
	OEA	.07	.05	2.68					
Model 3					378.40	.83	.70	0	.69
	SEA	.80	1.20	29.20					
	OEA	.06	.04	2.09					
	UOE	.05	.05	1.64					
Model 4					283.75	.83	.70	0	.69
	SEA	.81	1.22	26.62					
	OEA	.06	.05	2.19					
	UOE	.05	.06	1.68					
	ROE	-.02	-.04	-.80					
Model 5					226.67	.84	.70	0	.69
	SEA	.81	1.22	26.07					
	OEA	.60	.05	2.18					
	UOE	.05	.06	1.70					
	ROE	-.02	-.04	-.81					
	Gender	.01	.02	.44					

*CAAS* Career adapt-abilities scale, The CAAS subscales are concern, control, curiosity, and confidence, *WLEIS* Wong and law emotional intelligence scale. The WLEIS subscales are *SEA* Self-emotion appraisal, *OEA* Other people's emotion appraisal

*N* = 500

^*^p < .05. **p < 0.01. ***p < .001

For concern in model 1, students’ SEA was entered into the equation, *F*(1, 498) = 58.64, *p* < .001, accounting for 11% of the variance. In Model 2, students’ OEA was entered into the equation, *F*(2, 497) = 35.59, *p* < .001, explaining an additional 2%. In model 3, students’ UOE was entered into the equation, *F*(3, 496) = 23.68, *p* <.001, indicating no change in variance. In model 4, students’ ROE was entered into the equation, *F*(4, 495) = 17.73, *p* <.001, indicating no change in variance. In model 5, students’ gender was entered into the equation, *F*(5, 494) = 14.18, *p* <.001, indicating no change in variance. In model 6, students’ religious belief/s was entered into the equation, *F*(6, 493) = 11.80, *p* <.001, indicating no change in variance.

For control, in model 1, students’ SEA was entered into the equation, *F*(1, 498) = 147.428, *p* < .001, accounting for 23% of the variance. In Model 2, students’ OEA was entered into the equation, *F*(2, 497) = 88.05, *p* < .001, explaining an additional 3.3%. In model 3, students’ UOE was entered into the equation, *F*(3, 497) = 58.59, *p* <.001, indicating no change in variance. In model 4, students’ ROE was entered into the equation, *F*(4, 496) = 44.19,* p* <.001, indicating no change in variance. In model 5, students’ gender was entered into the equation, *F*(5, 494) = 35.54, *p* <.001, indicating no change in variance. In model 6, students’ religious belief/s was entered into the equation, *F*(6, 493) = 29.60, *p* <.001, indicating no change in variance.

For curiosity, in Model 1, students’ SEA was entered into the equation, *F (*1, 498) = 42.84, *p* < .001, 8.0% of the variance. In Model 2, students’ OEA was entered into the equation, *F*(2, 497) = 38.46, *p* < .001, an additional 6.0%. In model 3, students’ UOE was entered into the equation, *F*(3, 497) = 25.83, *p* <.001, indicating no change in variance. In model 4, students’ ROE was entered into the equation, *F*(4, 496) = 19.34, *p* <.001, indicating no change in variance. In model 5, students’ gender was entered into the equation, *F*(5, 494) = 16.49, *p* <.001, indicating no change in variance. In model 6, students’ religious belief/s was entered into the equation, *F*(6, 493) = 13.71, *p* <.001, indicating no change in variance.

For confidence, in Model 1, students’ SEA was entered into the equation, *F*(1, 498) = 1107.67, *p* < .001, explaining 69% of the variance. In Model 2, students’ OEA was entered into the equation, *F*(2, 497) = 564.33, *p* < .001, an additional 0.4%, are presented in Table 6. In model 3, students’ UOE was entered into the equation, *F*(3, 497) = 378.40,* p* <.001, indicating no change in variance. In model 4, students’ ROE was entered into the equation, *F*(4, 496) = 283.75, *p* <.001, indicating no change in variance. In model 5, students’ gender was entered into the equation, *F*(5, 494) = 226.67, *p* <.001, indicating no change in variance. In model 6, students’ religious belief/s was entered into the equation, *F*(6, 493) = 193.09, *p* <.001, indicating no change in variance.

## Discussion

University graduates go through substantial changes as they make the transition from being students to becoming working adults [54]. The current study examines whether emotional intelligence is associated with career adaptability during this period. Chinese youths were asked about their concern, control, curiosity and confidence with respect to career adaptability as well as their emotional intelligence in the domains of self-emotion appraisal (SEA), others’ emotion appraisal (OEA), use of emotion (UOE) and regulation of emotion (ROE). The results of this cross-sectional study reveal statistically positive and significant relationships between emotional intelligence traits and the multiple dimensions of career adaptability. The results are consistent with other research on this topic [4, 16, 19, 28, 50, 55–58] in indicating emotional intelligence to be a good predictor of career adaptability. This is the only study in the Asia-Pacific region to provide evidence of the links between emotional intelligence and career adaptability in Chinese youths.

### Self-emotion appraisal and other people's emotion appraisal in relation to career adaptability

The self-emotion appraisal and other people's emotion appraisal domain of emotional are associated with all four domains of career adaptability. The findings reveal that youths with keener observation of their own and others’ emotion report higher levels of career adaptability in all four domains.

Our findings are similar to those of recent research shows the mediating role of the link between emotional intelligence and career-related outcomes such as academic satisfaction [28], career decisions, and self-perceived employability [50].

Our results also support Brown et al.'s [16] view that people who can label their emotions effectively, understand complex feelings, and remain open to their own and others' pleasant and unpleasant feelings, demonstrate a high degree of clarity, enthusiasm and confidence in their commitment to career choices. Thus, they look enthusiastically for any opportunity in career-related tasks to satisfy their interests, values, and desires [4, 16].

Our findings are consistent with those of Gardner and Goleman et al. [59], who report that people who are effective workers with leadership traits, especially business leaders, are aware of and understand their own and other’s emotions. Previous studies [60–62] have found a positive correlation between trait emotional intelligence and transformational leadership, which emphasizes intrinsic motivation, others’ emotions, and positive development [63].

Transformational leaders are well equipped to make decisions and take responsibility for their actions in different workplace scenarios [16]. They also carefully observe different ways of doing things, and probe deeply into questions because they can observe and understand the emotions of others around them [62].

Our findings are backed by those of Coetzee & Schreuder [64], who find that understanding one’s own and others’ emotions predicts employability satisfaction and career adaptability. Employability satisfaction indicates the ability to establish positive relationships with others and obtain satisfaction, happiness, and self-assurance from serving others [64, 65]. For example, a young person who lacks awareness of thier own emotions and the emotions of others can easily show a lack of confidence in his employability [64].

Our findings are consistent with those of Brown et al. [16], who find that emotionally stable people have high confidence in their abilities to perform career-related tasks successfully. By understanding and expressing their emotions, people can solve problems independently [4] because they realize their strengths and know how to overcome their weaknesses by collaborating with others at work [16].

### Use of emotion and regulation of emotion in relation to career adaptability

Youths with more skilled use and higher regulation of emotion report higher levels of all domains of career adaptability. Our findings, consistent with those of Parmentier et al. [57] and Rudolph et al. [66], show that people with higher ability in managing emotion spontaneously work hard, plan their career in advance, and set the goals they want to achieve. Individuals who are good at using emotions are likely to have self-discipline, strong reactivity, and higher autonomy [16].

Our findings correspond with those reported by Rudolph et al. [66]: that the positive effects of emotional intelligence and career adaptability are due to the effect of self-regulation on self-efficacy with regard to career decision-making. In order words, people with high self-regulation and self-efficacy are likely to hold on their dreams and goals, and to go forward courageously once they believe they are right.

Mittal [67] finds that the use and regulation of emotions play a significant role in shaping career adaptability, satisfaction, and job search success. The ability to use and regulate emotions create self-regulating psychological resources, which has a positive and constructive impact on job search success [4, 66].

Coetzee and Harry's [19] study further indicates that the ability to manage one's own emotions, and career concern, are important psychosocial career meta-capacities that predict career adaptability. Similarly, Gardner and Stough [60] find that effective and successful leaders are able to recognize and apply their own and others' emotional knowledge and the ability to control emotional states when interacting and solving problems.

Our findings are supported by research conducted by Parmentier et al. [57], who indicate that individuals who are well controlled emotionally are more interested in looking for new career opportunities. While career adaptability is considered a self-regulating resource, career adaptability will change over time, along with the individual's changing regulation needs [68–70]. It is recommended that young professionals incorporate emotional intelligence into the career development process [4].

Our findings are also consistent with those reported by Brown et al. [16], who show that self-motivated and emotionally self-regulated individuals demonstrate preparation and confidence in the process of career decision-making and implementation. Adolescents with these characteristics can solve problems and overcome obstacles independently because they are relatively mature and autonomous. Emotional intelligence is an important meta-ability that can cultivate adaptive behaviors and ultimately promote personal adaptation to professional career challenges and personal-self-assurance [4].

Emotions help people complete tasks smoothly and on time, because people have a better understanding and management of their own emotional reactions and are thus not so easily affected by emotions [29, 71].

## Limitations

The study has three major limitations. Firstly, the generalizability of the findings may be limited because the sample of 500 young people from two universities is relatively small compared with the total population of Chinese youths in Hong Kong. Future studies should seek to replicate these results in a sample of Chinese youth in cities such as Shanghai and Shenzhen.

Secondly, although the WLEIS and CAAS have been shown to be reliable and valid multi-dimensional measures of emotional intelligence and career adaptability respectively, there is still a lack of agreement in the literature about the meaning of terms and concepts such as adaptability, regulation of emotion and self-emotion appraisal. Future research should consider collecting additional reports from third parties, such as parents and teachers. Also, future studies should consider using these instruments simultaneously with qualitative studies, such as in-depth interviews and focus group discussions, as a mixed research methodology for the purpose of triangulation. This will not only help verify and enrich our findings but also address this limitation.

Thirdly, regarding the WLEIS, participants might not be accurate reporters of their emotional status. As the respondents may tend to select the ideal answers (i.e., preference and the best one) instead of their real answers (experiences) owing to social expectations, they may well overrate or underrate themselves in the questionnaire. Also, the measure does not include a validity scale to check for inconsistent answers. Owing to the cross-sectional design of our study, the hypothesis of causation should be considered carefully. As follow-up longitudinal studies will help address this limitation, future studies using a prospective design are needed to confirm our findings.

Finally, the findings may not be more comprehensive because only students from the Business discipline were recruited. Students from other academic disciplines, such as Science, Social Science, Art, and Humanities should be recruited in the future samples. This would provide more comprehensive representation and deeper analysis of the youths.

### Implications for 1) research, and 2) practice

Future studies should replicate these results in Chinese youth elsewhere in China, through longitudinal designs or experimental studies, to investigate causal relationships between variables. Future studies should further explore through qualitative methodology, how and why emotional intelligence and career adaptability are related. Future research could be more specific and in-depth, examining, for example, whether the relationships and predicted values are the same across industries.

The results have implications for further study exploring whether emotional intelligence incorporated into the curriculum design, would better prepare students for future work-related changes. It would be valuable to be conducted more such studies in the future. For example, experiential learning, including community service learning [72] and work-integrated learning [73], could also be considered as avenues through which young people may improve their emotional intelligence and career adaptability. In addition, aptitude tests should be administered to adolescents to fully reveal their potential and talents in their career paths. The current research also offers unique insights for educators and teachers responsible for well-rounded development of students, thereby fostering healthy lifestyles, stable emotional well-being and greater career resilience in adolescents.

## Conclusion

The findings of this study suggest that Chinese youth graduates with high trait emotional intelligence are also likely to show more concern, control, curiosity, and confidence regarding career adaptability. All domains of emotional intelligence (SEA, OEA, UOE, and ROE) are associated with higher career adaptability, but the link is especially strong for the SEA domain. There have been similar studies in the west, such as Parmentier et al. [4] in Belgium, and Udayar et al. [50] in Switzerland but this is the first study on the trait emotional intelligence of Chinese youths in the Asia-Pacific region. The results may inform career development interventions for youth professionals.

## Data Availability

The analysis results generated in the current study are presented in this published article. The research datasets will not be publicly available because they contain sensitive material, identifying participant information. The datasets generated for this study are available from the corresponding author on reasonable request.

## Abbreviations

EI: Emotional intelligence
EP: Emotional perception
MOE: Managing others' emotions
SEA: Self-emotion appraisal
OEA: Other people's emotion appraisal
ROE: Regulation of emotion
UOE: Use of emotion
